# Nocturnal versus diurnal CO_2_ uptake: how flexible is *Agave angustifolia*?

**DOI:** 10.1093/jxb/eru097

**Published:** 2014-03-19

**Authors:** Klaus Winter, Milton Garcia, Joseph A. M. Holtum

**Affiliations:** ^1^Smithsonian Tropical Research Institute, PO Box 0843-03092, Balboa, Ancon, Republic of Panama; ^2^Tropical Biology, James Cook University, Townsville, Queensland 4811, Australia

**Keywords:** *Agave*, biofuel, climate change, crassulacean acid metabolism, C_3_ photosynthesis, CO_2_ response, drought stress, temperature response.

## Abstract

Agaves exhibit the water-conserving crassulacean acid metabolism (CAM) photosynthetic pathway. Some species are potential biofuel feedstocks because they are highly productive in seasonally dry landscapes. In plants with CAM, high growth rates are often believed to be associated with a significant contribution of C_3_ photosynthesis to total carbon gain when conditions are favourable. There has even been a report of a shift from CAM to C_3_ in response to overwatering a species of *Agave*. We investigated whether C_3_ photosynthesis can contribute substantially to carbon uptake and growth in young and mature *Agave angustifolia* collected from its natural habitat in Panama. In well-watered plants, CO_2_ uptake in the dark contributed about 75% of daily carbon gain. This day/night pattern of CO_2_ exchange was highly conserved under a range of environmental conditions and was insensitive to intensive watering. Elevated CO_2_ (800 ppm) stimulated CO_2_ fixation predominantly in the light. Exposure to CO_2_-free air at night markedly enhanced CO_2_ uptake during the following light period, but CO_2_ exchange rapidly reverted to its standard pattern when CO_2_ was supplied during the subsequent 24h. Although *A. angustifolia* consistently engages in CAM as its principal photosynthetic pathway, its relatively limited photosynthetic plasticity does not preclude it from occupying a range of habitats, from relatively mesic tropical environments in Panama to drier habitats in Mexico.

## Summary

In young and mature plants of the CAM species *Agave angustifolia*, the day/night pattern of CO_2_ exchange was highly conserved under a range of environmental conditions.

## Introduction

Most, if not all, of the approximately 200 species in the New World genus *Agave* (family Asparagaceae, subfamily Agavoideae; Chase *et al.*, 2009; Govaerts *et al.*, 2013) exhibit the nocturnal uptake of CO_2_ and accumulation of malic acid characteristic of crassulacean acid metabolism (CAM) (Nobel, 2003, 1996*a*). In addition to a water-use-efficient carbon metabolism, these archetypal dry-land CAM plants sport a xerophytic, water-conserving morphology that includes succulent, angled, persistent leaves with thick cuticles, sunken stomata, and a rosette leaf configuration around a central stem that channels rain water and condensate to the base of the roots (Gentry, 1982).

As biomass becomes an increasingly valuable commodity for energy generation, there is realization that the seasonally dry or semi-arid landscapes inhabited by *Agave*, whilst not optimal for growing traditional water-demanding food crops, may nonetheless be suitable for biomass generation (Borland *et al.*, 2009; Chambers and Holtum, 2010; Davis *et al.*, 2011; Holtum *et al.*, 2011; Yan *et al.*, 2011). In such landscapes, water-use-efficient *Agave* can accumulate biomass at annual rates that approach those produced by C_4_ plants like sugar cane and *Miscanthus* in higher rainfall regions (Nobel, 1991, 1996*a*; Somerville *et al.*, 2010). For example, productivities of 25, 35, and 47–50 Mg dry weight ha^–1^ year^–1^ have been reported for the CAM species *Agave tequilana*, *Ananas comosus* (pineapple) and *Opuntia ficus-indica*, respectively (Nobel, 1996*a*). *A. tequilana* is now being trialed in the seasonally dry Australian tropics as a biofuel feedstock (Chambers and Holtum, 2010; Holtum *et al.*, 2011).

The extent and capacity for day-time CO_2_ fixation in well-watered agaves tends to be limited, but its expression differs among species (see Nobel, 2003, for a review). Interestingly, well-watered plants of mature *Agave deserti* did not exhibit afternoon CO_2_ fixation, but when ‘overwatered’ (watered daily for 10 weeks) they switched to overwhelmingly C_3_ photosynthesis (Hartsock and Nobel, 1976).

Using continuous whole-plant gas exchange, we explored here the potential for photosynthetic plasticity in *Agave angustifolia*, a putative wild ancestor of the agronomically significant species *Agave fourcroydes* and *A. tequilana* (Gentry, 1982; Colunga-García Marín *et al.*, 1999). *A. angustifolia* was chosen because, across its range from Mexico to Panama, it is found in habitats as diverse as coastal dunes at sea level to oak-pine forests at 2200 m (García-Mendoza and Chiang, 2003), and it grows in Panama (the site of this study) in relatively mesic environments where CO_2_ fixation in the light is expected to be more favoured than in drier habitats.

In order to determine photosynthetic pathway plasticity of *A. angustifolia*, we examined 24 plants. Our goal was to explore conditions under which plants would markedly upregulate C_3_ photosynthetic CO_2_ uptake in the light. In two young plants, net CO_2_ exchange was continuously monitored for 234 and 281 day/night cycles, during which the plants were exposed to a range of perturbations (light, temperature, CO_2_, and watering regime) that have been reported to affect CO_2_ uptake in the light in other CAM plants. Furthermore, net CO_2_ exchange was monitored in two extremely well-watered mature plants in a naturally illuminated gas-exchange chamber for up to 16 d each, in order to document the effect of natural day-to-day variation in photon flux density (PFD) on light and dark CO_2_ fixation. Thirdly, a total of 20 young, well-watered plants were grown under two CO_2_ concentrations and two nutrient regimes to manipulate the relative contributions of day and night CO_2_ fixation to growth. Although the longer than 200 d gas-exchange experiments were not replicated in the strictest sense because of their duration, all experiments taken together permitted a reasonable assessment of the degree of phenotypic photosynthetic plasticity in *A. angustifolia*. The results indicated that *A. angustifolia* exhibits a conserved carbon fixation strategy rather than using the light and dark options of CO_2_ uptake in a highly flexible manner.

## Materials and methods

### Plant material

*A. angustifolia* Haw. was collected from Playa Majagual, Panamá (8°43′N, 79°45′E), and grown outdoors in forest topsoil at the Smithsonian Tropical Research Institute, Santa Cruz Experimental Research Facility, Gamboa, Republic of Panama (9°07′N, 79°42′W). Opinions differ as to whether *A. angustifolia* is a synonym of *Agave vivipara* L. or whether the two are distinct species (Wijnands, 1983; García-Mendoza and Chiang, 2003; Govaerts *et al.*, 2013). Vouchers of the *Agave* examined in this study were deposited in the herbarium of the University of Panama (JARANDA 4484A and 4484B).

### Measurement of CO_2_ exchange in the laboratory

Bulbils of between 5 and 10cm in height, comprising two to three leaves, were enclosed in a Perspex cuvette (internal dimensions 11×11×10 or 20×20×15cm) by passing the base of a plantlet through a hole in the cuvette base and sealing the plantlet–cuvette interface with a non-porous synthetic rubber sealant (Terostat VII; Henkel-Teroson, Heidelberg, Germany). The root-containing base of the plantlet outside the cuvette was planted in a 1 litre pot containing potting mix (Cactus, Palm and Citrus Soil; Miracle-Gro Lawn Products, Marysville, OH, USA) and 2g of Osmocote Plus fertilizer (Scotts-Sierra Horticultural Products, OH, USA).

The gas-exchange cuvette was located inside a controlled-environment chamber (Environmental Growth Chambers, OH, USA) operating under 12h light (28 °C)/12h dark cycles (17 or 22 °C as specified). PFD was measured at the top of the cuvette. Air containing 200, 400, or 800 ppm CO_2_ was generated by a CO_2_/CO_2_–free-air mixing unit (Walz GmbH, Effeltrich, Germany). Net CO_2_ exchange of plantlets was measured in flow-through gas-exchange systems consisting of Walz components and LI-6252 CO_2_ analysers (Li-Cor, Lincoln, NE, USA) (Holtum and Winter 2003). Normal watering involved supplying water at least once every second day, and intensive watering involved supplying water twice per day. Drought treatments were imposed by withholding irrigation altogether.

### Measurement of whole-plant CO_2_ exchange under natural light

A mature plant, established in forest topsoil containing 50g of Osmocote Plus fertilizer (Scotts-Sierra Horticultural Products), in a 190 l pot was placed inside a ventilated, naturally illuminated chamber constructed of glass panels and an aluminium framework (internal volume 8.8 m^3^). A blower (model 4C054; Grainger Industrial Supply, OH, USA) supplied external air to the chamber at 10.5 m^3^ min^–1^. Within the chamber, air was circulated by four fans and temperature was regulated by a split air-conditioning system (model V1124C2H; Innovair, FL, USA). Whole-plant gas exchange was quantified at 30min intervals from the rate at which the CO_2_ concentration inside the chamber changed when air flow into the chamber was blocked for 5min, thereby converting the chamber into a closed system. Changes in the CO_2_ concentration inside the chamber were measured using a LI-7500 open-path CO_2_ analyser (Li-Cor). Calculations of net CO_2_ exchange were based on chamber volume that had been corrected for the volumes of the pot, plant, and equipment inside the chamber, and the rate at which the CO_2_ concentration changed during the period when the chamber was isolated. CO_2_ measurements were corrected for changes of temperature and humidity. Measurements of PFD were taken outside the chamber. PFD inside the chamber was approximately 15% below that outdoors. For further details of methods, see Winter *et al.* (2009). The experiment was repeated for a second mature plant (data not shown).

### Growth of plants at 280 and 800 ppm CO_2_

Twenty plants in 19 l pots were grown with daily watering in forest topsoil for 166 d inside two naturally illuminated glasshouses (internal volume 37.8 m^3^ each). Five of the 10 pots in each chamber were supplemented with 8g of Osmocote Plus fertilizer (Scotts-Sierra Horticultural Products). One glasshouse was maintained at 280±10 ppm (range) CO_2_ by passing chamber air through soda lime to lower [CO_2_]. An above-ambient CO_2_ concentration of 800±10 (range) ppm was achieved in the second glasshouse by releasing pulses of CO_2_ gas into the chamber from a high-pressure cylinder in conjunction with a feedback control system. Within each glasshouse, air was circulated by five fans, and a split air-conditioning system maintained temperatures at close to ambient (Cernusak *et al.*, 2011).

### Titratable acidity

Leaf discs punched from the centre of fully expanded leaves using a cork borer at the end of the light and dark periods were frozen in liquid nitrogen. Organic acids were extracted by sequentially boiling samples in 50% ethanol and water for 5min. Extracts were cooled to room temperature and titrated with 10mM KOH to pH 6.5 (Holtum *et al.*, 2004).

### Stable isotope analysis

The δ^13^C values of finely ground homogenous powder from the pooled dried leaves of whole plants were measured in an isotope ratio mass spectrometer (Delta V; Thermo Fisher Scientific) in the Stable Isotope Laboratory of the Smithsonian Tropical Research Institute. The abundance of ^13^C in each sample was calculated relative to the abundance of ^13^C in standard CO_2_ that had been calibrated against Pee Dee belemnite (*Belemnitella americana*). Relative abundance was determined using the relationship:

$$\delta^{\text{13}}\text{C }\left(‰\right)=\left[\left({}^{\text{13}}\text{C}{/}^{\text{12}}\text{C of sample}\right)/\left({}^{\text{13}}\text{C}{/}^{\text{12}}\text{C of standard}\right)–\text{1}\right]\times \text{1}000.$$

The δ^13^C values of two C_4_ plant species, *Saccharum spontaneum* and *Portulaca oleracea*, grown in each glasshouse and outside in the open air were used to correct for differences in the δ^13^C value of the source CO_2_ (Cernusak *et al.*, 2011). The CO_2_ purchased for CO_2_ enrichment was from a natural CO_2_ spring and a correction of 2‰ was applied.

## Results

**Fig. 1.**
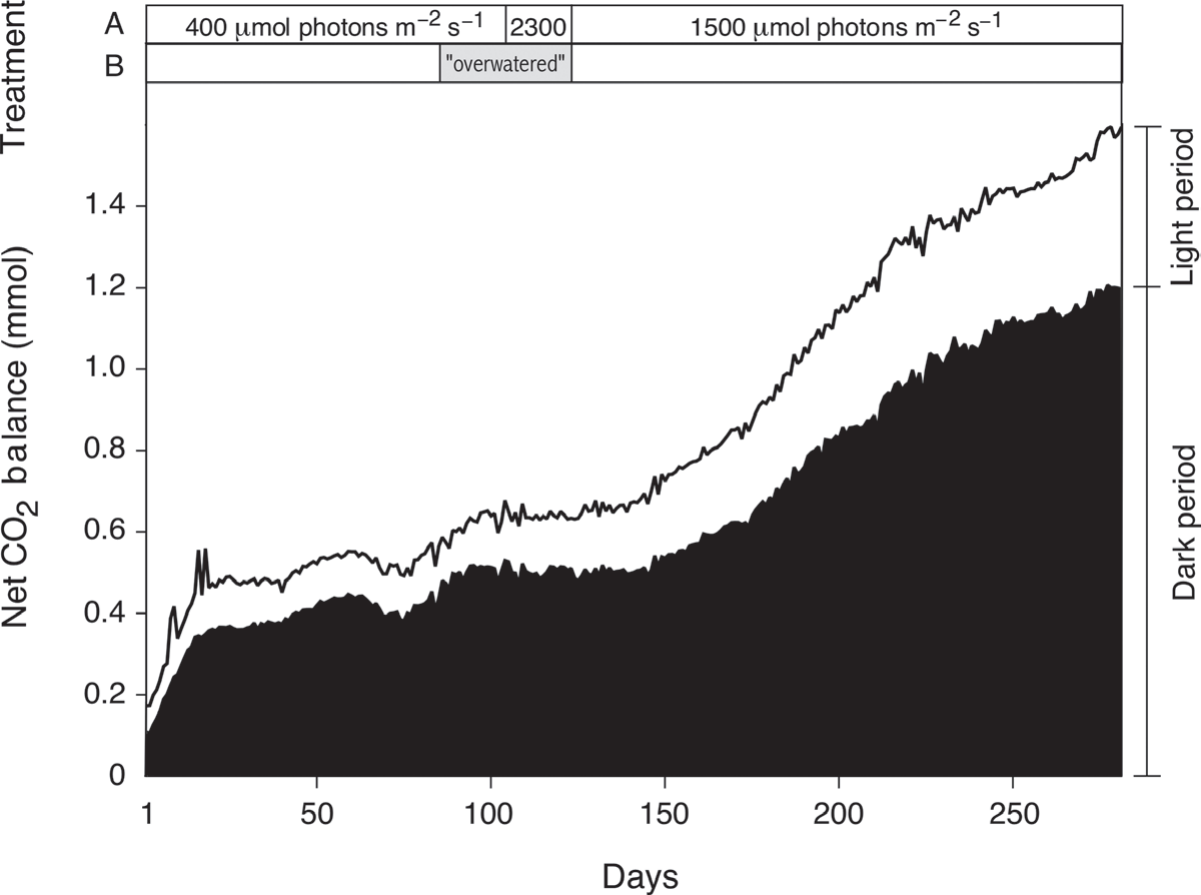
Net CO_2_ balance of the shoot of a young individual of *A. angustifolia* during 281 consecutive 12h light/12h dark cycles. Carbon balances are shown for the light and dark periods. The following treatments were applied: (A) PFD was 400 µmol photons m^–2^ s^–1^ during the initial 103 d, 2300 µmol photons m^–2^ s^–1^ between d 104 and 122, and 1500 µmol photons m^–2^ s^–1^ thereafter. (B) Irrigation was increased from once every 2 d to twice per day between d 85 and 122 (‘overwatered’).

CO_2_ fixation in the dark (CAM phase I; Osmond, 1978) contributed 78% of the carbon gain in a young well-watered *A. angustifolia* for which CO_2_ uptake was monitored continuously during 281 consecutive 12h light/12h dark cycles (Fig. 1). The predominance of dark fixation was maintained under 12h PFDs of 400 μmol m^–2^ s^–1^ (17.3mol m^–2^ d^–1^), 1500 μmol m^–2^ s^–1^ (64.8mol m^–2^ d^–1^), and 2300 μmol m^–2^ s^–1^ (99.4mol m^–2^ d^–1^).

**Fig. 2.**
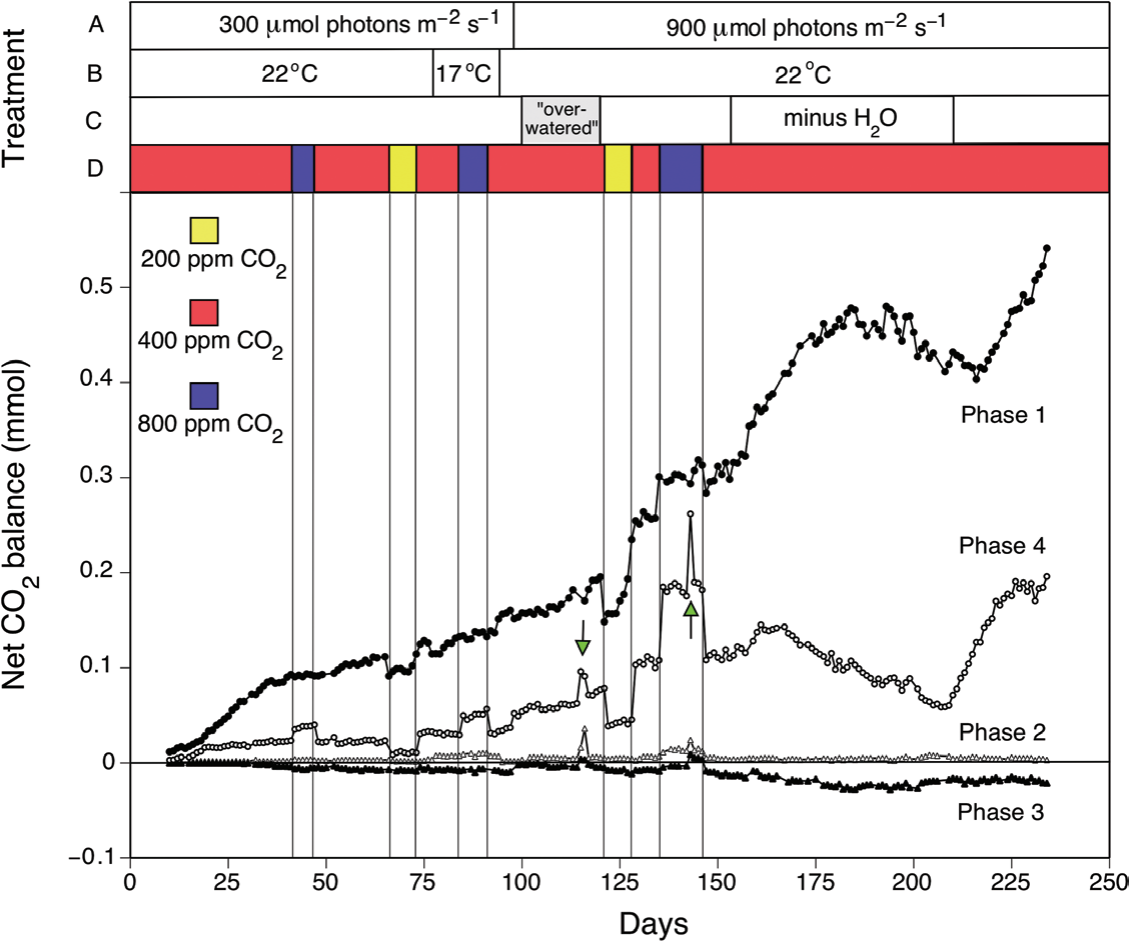
Net CO_2_ balance of the shoot of a young individual of *A. angustifolia* during 234 consecutive 12h light/12h dark cycles. Carbon balances are shown for the four phases of the 24h CAM cycle. As indicated by the four horizontal bars in the upper part of the figure, the following treatments were imposed in the course of the experiment: (A) PFD was 300 µmol photons m^–2^ s^–1^ up to d 98, and increased to 900 µmol photons m^–2^ s^–1^ thereafter. (B) Temperature during the dark period was lowered from 22 °C (standard night-time condition) to 17 °C between d 77 and 93. (C) Irrigation was stopped between d 153 and 209, and irrigation was increased from once to twice per day between d 100 and 121 (‘overwatered’). (D) Ambient [CO_2_] was 200, 400, or 800 ppm as indicated by yellow, red, and blue, respectively. The two green arrows indicate net CO_2_ exchange in the light period following exposure of the plant to CO_2_-free air during the previous night (d 114, 115, and 142). Data for the initial 9 d of the experiment are not shown. (This figure is available in colour at *JXB* online.)

Uptake of CO_2_ in the dark remained the principal contributor to net carbon gain during 234 day/night cycles of gas exchange when a young *A. angustifolia* was subjected to 300 or 900 μmol m^–2^ s^–1^ light; 200, 400 or 800 ppm CO_2_; when well-watered, overwatered, or non-watered; and when night temperatures were 17 or 22 °C (Fig. 2). CO_2_ uptake in the afternoon (CAM phase IV) was also always present, whereas the contribution of dawn CO_2_ fixation (CAM phase II) to plant carbon gain was minimal. Net CO_2_ loss was consistently observed during midday stomatal closure (CAM phase III) except for small CO_2_ gains on those days when CO_2_ was withheld during the dark while the plant was exposed to 800 ppm CO_2_. Over the course of the experiment (Fig. 2), the contributions to net carbon gain by phases I, II, III, and IV were 78.5, 1.7, –4.2, and 24%, respectively.

The contributions of light and dark CO_2_ uptake to net carbon gain under 200 or 800 ppm CO_2_ differed (Fig. 2). When the CO_2_ concentration was reduced from 400 to 200 ppm, CO_2_ uptake at a PFD of 300 μmol m^–2^ s^–1^ was reduced by 62% during phase IV and by 16% in the dark. The reductions were 56 and 25%, respectively, at 900 μmol m^–2^ s^–1^. In contrast, when the atmospheric CO_2_ concentration was increased from 400 to 800 ppm, CO_2_ uptake by the plant at 300 μmol m^–2^ s^–1^ increased by 64% during phase IV and remained unchanged during phase I. At a PFD of 900 µmol m^–2^ s^–1^, the increases in CO_2_ uptake during phases IV and I were 73 and 10%, respectively

Withholding water from the plant for 54 d also differentially influenced CO_2_ fixation in the light and in the dark (Fig. 2). Overall net carbon gain by the plant was initially stimulated but was subsequently reduced as water in the pot became limiting. During the initial 34 d without watering, as CO_2_ uptake increased by 46% in the dark and decreased by 17% during phase IV in the light, the contribution of nocturnal uptake to net carbon gain rose from 74 to 85%. Subsequently, CO_2_ exchange fell during the light and the dark. The decrease was proportionally less in the dark such that, at the end of the drought treatment, dark fixation contributed 88% to net carbon gain. In contrast to drought, intensive watering of the plant for 30 d did not affect the relative contributions of light and dark CO_2_ fixation to carbon gain, nor did decreasing the night temperature from 22 to 17 °C.

**Fig. 3.**
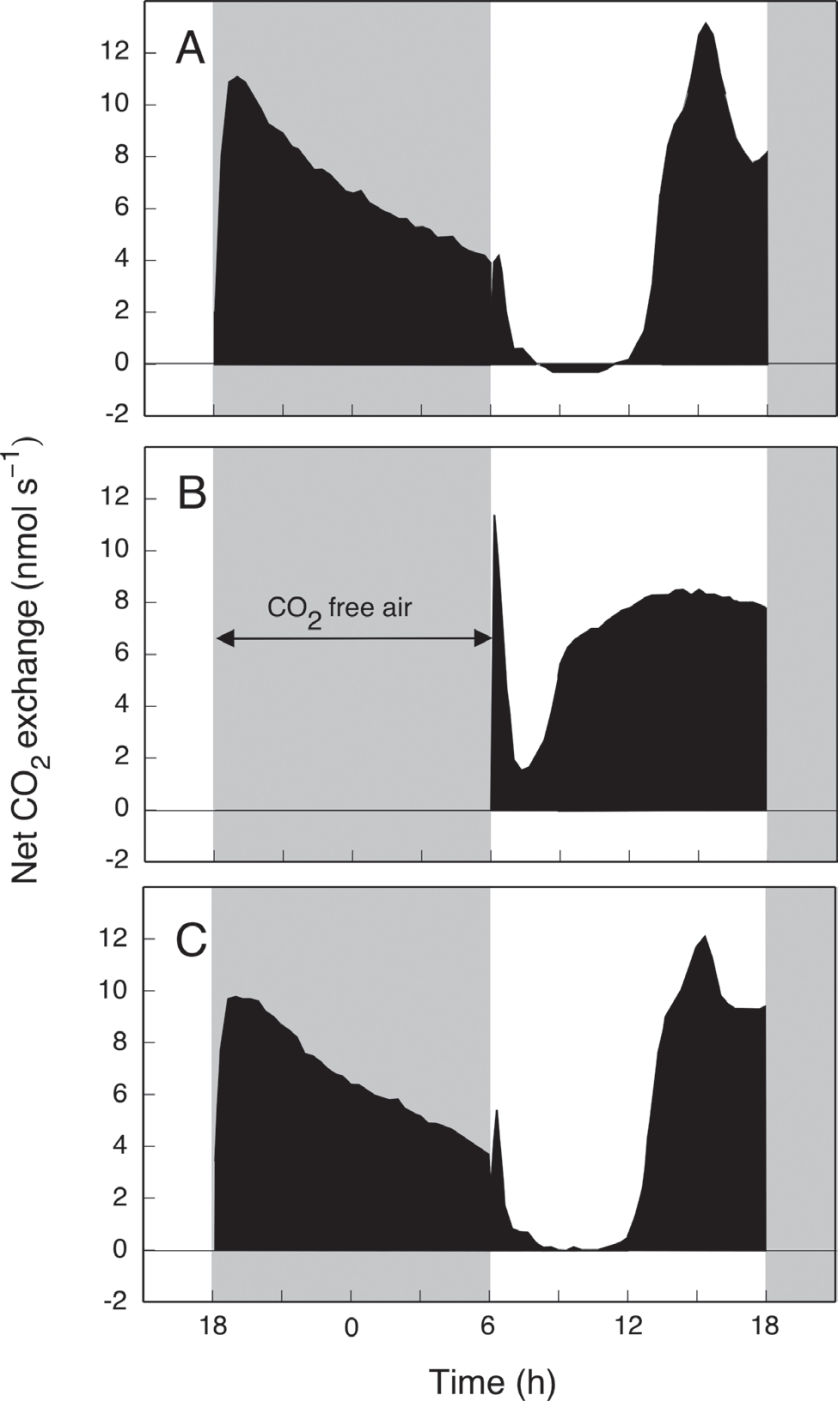
Gas exchange by a shoot of a well-watered young individual of *A. angustifolia* (d 141, 142 and 143 of the shoot shown in Fig. 2.) supplied with 800 ppm CO_2_ during the dark and during the light on d 141 (A), supplied with 0 ppm CO_2_ during the dark and 800 ppm CO_2_ during the light on d 142 (B), and supplied with 800 ppm CO_2_ during the dark and during the light on d 143 (C). Shading indicates the night. Day-time PFD was 900 µmol photons m^–2^ s^–1^.

CO_2_ assimilation in the light was upregulated when CO_2_ was removed from the air supply during the preceding dark period (Fig. 2, d 141 and 142). Fig. 3 details how both the extent and pattern of CO_2_ exchange in the light differed following exposure to CO_2_-free air during the night. CO_2_ was assimilated throughout the light principally via the contribution of an extended phase IV (Fig. 3B). Phase III was transient, and phase II did not increase in duration although the rate of CO_2_ uptake did increase. During the subsequent dark/light cycle, CO_2_ exchange returned to the patterns observed prior to the CO_2_-free treatment (Fig. 3C).

**Fig. 4.**
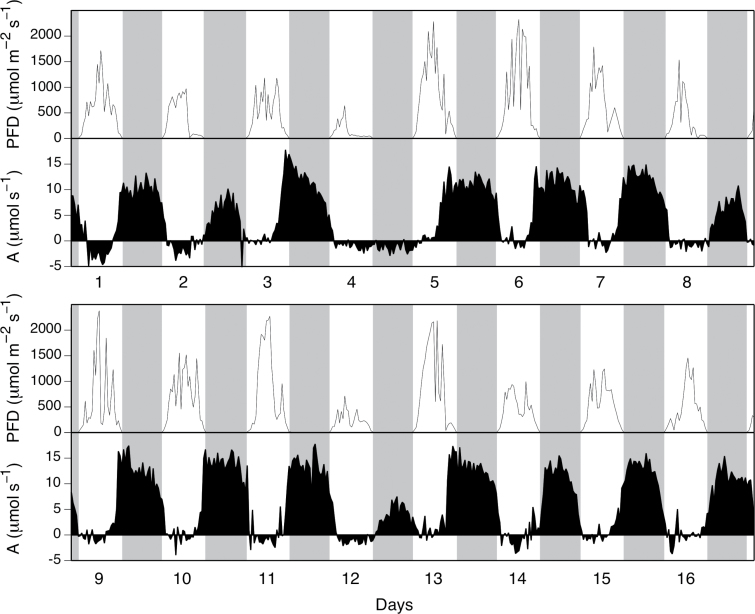
PFD (upper panels) and net CO_2_ exchange (lower panels) for the shoot (aboveground parts that included 51 leaves) of a mature *A. angustifolia* prior to the emergence of the reproductive stalk. Measurements were conducted from 27 July 2011 (d 1) to 11 August 2011 (d 16) in a naturally illuminated gas-exchange chamber. Shading indicates night.

Nocturnal CO_2_ uptake was the principal contributor to carbon gain in fully mature, well-watered *A. angustifolia* grown outdoors under natural sunlight (Fig. 4). Day-time CO_2_ uptake occurred mainly during the late afternoon (phase IV). The contribution of phase II CO_2_ uptake was variable but generally small, whereas net CO_2_ loss was consistently observed during phase III midday stomatal closure.

Nocturnal CO_2_ uptake decreased substantially following overcast days (Fig. 4) For example, the extremely overcast d 4 light period was followed by a night during which there was no net CO_2_ uptake. Less extreme examples of this trend were evident on d 2, 8, and 12. On days following overcast days, CO_2_ uptake during the afternoon tended to be more pronounced.

**Fig. 5.**
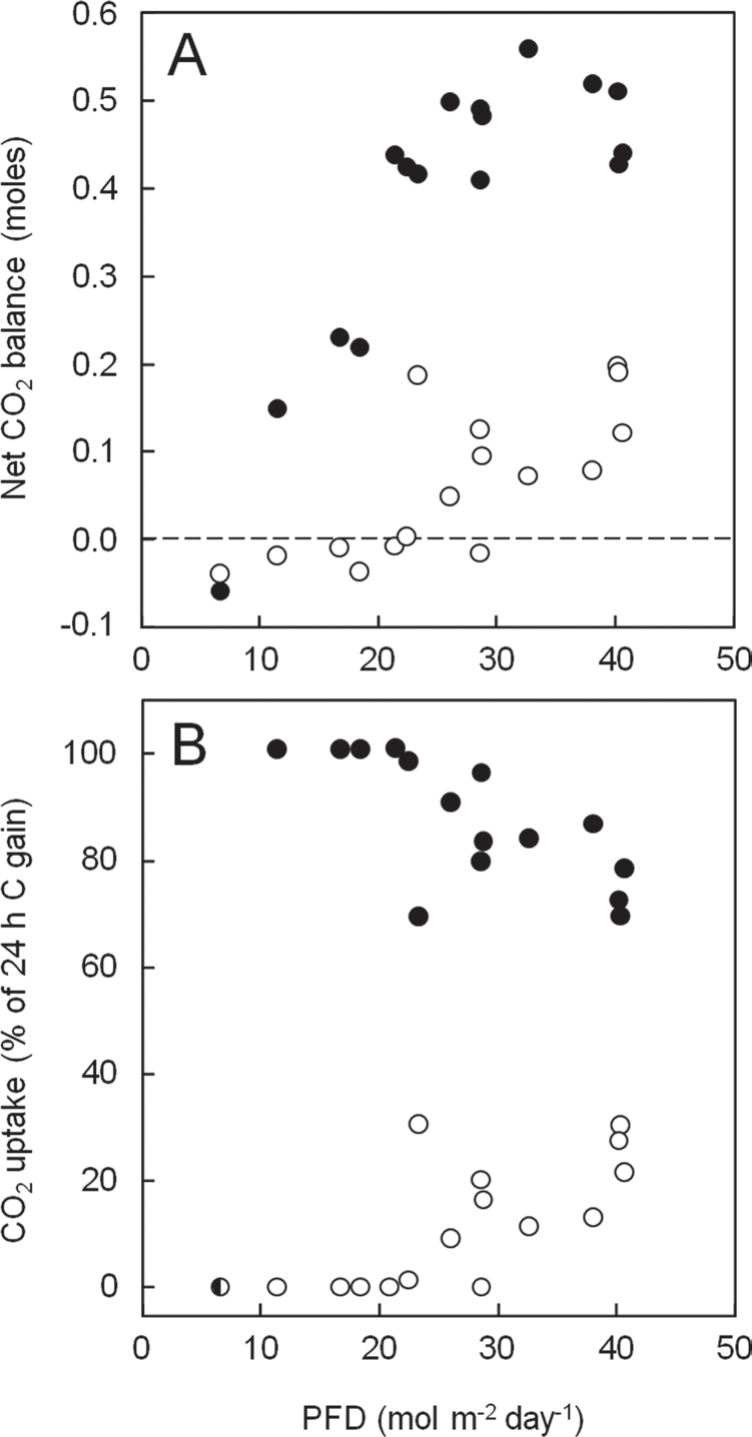
(A) Day-time (open symbols) and night-time (closed symbols) CO_2_ balances in response to daily integrated PFD of the mature *A. angustifolia* plant studied in Fig. 4. (B) Percentage contribution of day-time (open symbols) and night-time (closed symbols) CO_2_ uptake to 24h carbon gain. The symbol with open and closed halves represents d 4, an overcast day during which CO_2_ balances during the light and the dark were negative.

Fig. 5 quantifies the relationships between day-time and night-time CO_2_ exchange and daily PFD for the mature *A. angustifolia* illustrated in Fig. 4. Nocturnal CO_2_ uptake was correlated with the integrated PFD during the preceding light period and contributed predominately to net CO_2_ uptake at all light intensities that supported positive daily carbon gain. Following sunny days, when the integrated PFD exceeded about 30mol m^–2^ d^–1^, CO_2_ uptake at night was saturated, providing 70–85% of the daily carbon gain. Below 30mol m^–2^ d^–1^, total carbon gain fell and the proportional contribution of nocturnal CO_2_ uptake to 24h carbon gain rose. Day-time CO_2_ exchange became negative at around 21mol m^–2^ d^–1^, whereas night-time CO_2_ exchange became negative at about 10mol m^–2^ d^–1^.

The biomass of *A. angustifolia* grown in unfertilized soil at 800 ppm CO_2_ was double that of plants grown at 280 ppm CO_2_, whereas leaf acidities were similar on both mass and leaf-area bases (Table 1). Fertilization increased the biomass 2.5-fold in plants grown at 280 ppm CO_2_ and 1.7-fold in plants grown at 800 ppm CO_2_. For both unfertilized and fertilized treatments, the δ^13^C values for plants at 800 ppm CO_2_ were 1.8‰ more negative than for plants grown at 280 ppm CO_2_. In comparison to unfertilized plants, fertilized plants exhibited greater nocturnal accumulation of H^+^ per unit leaf area, but H^+^ accumulation per unit leaf mass was unchanged.

**Table 1.** Dry mass, nocturnal increase in leaf tissue acidity, and δ^13^C value (whole shoot) of A. angustifolia grown at 280 and 800 ppm CO_2_Values are means ±standard deviation (*n*=5), except for δ^13^C value at 280 ppm+fertilizer, where *n*=3. Means on the same row with different superscript letters differ significantly (two-way analysis of variance followed by Fisher’s least significant difference test, *P*<0.05).

Parameter	CO_2_ concentration			
	280 ppm		800 ppm	
	– fertilizer	+ fertilizer	– fertilizer	+ fertilizer
Total dry mass (g)	26±4^A^	65±12^B^	51±5^C^	88±11^D^
Nocturnal H^+^ increase:				
μmol g^–1^ fresh weight	226±40^A^	210±14^AB^	184±8^B^	207±31^AB^
μmol cm^–2^	79±12^A^	96±4^B^	77±6^A^	93±11^B^
δ^13^C (‰)	–14.6±0.2^A^	–14.0±0.2^B^	–16.4±0.2^C^	–15.8±0.2^D^

## Discussion

Despite an ability to occupy contrasting habitats, photosynthetic flexibility in *A. angustifolia* does not appear to be exceptional in terms of the proportional contributions to carbon gain of CO_2_ uptake in the dark and light. Nocturnal CO_2_ uptake was the principal source of carbon in mature *A. angustifolia* and in young plants once they had established. The proportional contribution to daily carbon gain of nocturnal CO_2_ uptake remained a remarkably consistent 70–85%, although the amount of CO_2_ fixed per day by well-watered plants varied with light intensity and nutrient status. In contrast to *A. deserti* (Hartsock and Nobel, 1976), the day/night pattern of CO_2_ exchange in *A. angustifolia* did not shift towards a C_3_ pattern when the supply of water was effectively unlimited.

Drought affected plant carbon gain and increased the proportional contribution of nocturnal CO_2_ uptake to it (Fig. 2). Two weeks after the cessation of irrigation, drought stress manifested itself as a continuous decline in light CO_2_ fixation. Most importantly, the initial 25 d of the decline of CO_2_ uptake in the light was accompanied by an increase in the rate of dark CO_2_ fixation. This drought-induced upregulation of CAM is a typical feature of facultative CAM. Facultative CAM or facultative components of CAM are not restricted to metabolically flexible annuals such as *Mesembryanthemum crystallinum* (Winter and von Willert, 1972) and *Calandrinia polyandra* (Winter and Holtum, 2011), and perennials such as some species of *Clusia* (Winter *et al*., 2009), but have also been observed in juveniles of constitutive CAM succulents such as *O. ficus-indica* and *Opuntia elatior* (Winter *et al*., 2008, 2011).

An attempt to force *A. angustifolia* into a C_3_-like photosynthetic pattern by exposing it to 800 ppm CO_2_ was partially successful in that daily carbon gain was enhanced and the proportional contribution of CO_2_ uptake in the light rose, for example from 29 to 40% during an 11 d treatment (Fig. 2). The ability to maintain this pattern of CO_2_ exchange was confirmed following a 166 d exposure to 800 ppm CO_2_ after which tissue δ^13^C values were close to those predicted by Winter and Holtum (2002) for a 40% contribution to carbon gain of CO_2_ fixation in the light.

In the short-term 800 ppm CO_2_ fumigation treatments, CO_2_ uptake in the dark was not or was only slightly enhanced (Fig. 2); in the longer-term experiment, there was no enhancement (Table 1), as nocturnal acidification remained unchanged. The contribution of nocturnal CO_2_ fixation to carbon gain in CAM tissues is variably responsive to environmental stimuli, which include night temperature, day-length, light intensity, and atmospheric CO_2_ concentration (Neales, 1973; Drennan and Nobel, 2000; Borland *et al.*, 2011). Increased CO_2_ assimilation in the light but not the dark has been reported in *Ananas* grown at 700 ppm CO_2_ under a 30/20 °C day/night regime that was optimal for nocturnal CO_2_ uptake at ambient CO_2_ (Zhu *et al.*, 1999). However, when *Ananas* was grown at higher night temperatures that were less than optimal for dark CO_2_ uptake at ambient CO_2_, the growth of plants at 700 ppm CO_2_ increased CO_2_ uptake in both the light and the dark. Increases in both light and dark CO_2_ fixation following exposure to high concentrations of atmospheric CO_2_ have been reported for *A. deserti* (Graham and Nobel, 1996) and *Agave salmiana* (Nobel, 1996*b*; Nobel *et al.*, 1996), and for the stem succulents, *O. ficus-indica* (Nobel and Israel, 1994) and *Stenocereus queretaroensis* (Nobel, 1996*b*).

*A. angustifolia* could be shifted towards a C_3_-like light-only CO_2_ uptake pattern by an extreme treatment that required withholding CO_2_ during the dark and supplying 800 ppm CO_2_ in the light (Fig. 3). In the light, the duration of phase IV increased at the expense of phase III, presumably because small amounts of acid formed at night would be rapidly consumed and the inhibition of stomatal opening by the resulting high internal CO_2_ concentration would be transitory. In the 24h cycle during which CO_2_-free air was supplied at night, daily carbon gain fell because the increase in CO_2_ gain in the light did not offset the lack of uptake of atmospheric CO_2_ during the night.

The stimulation of light fixation following exposure to CO_2_-free air at night lasted only during the light period following the treatment. Remarkably, no evidence of a metabolic memory of the CO_2_-free treatment was evident in the subsequent night and the day that followed it.

It has been suggested that the feasibility of cultivating strong-CAM plants such as *Agave* or *Opuntia* for biofuel feedstock in seasonally dry environments could be improved by developing plants that would fix a greater proportion of CO_2_ in the light during the moister parts of the year (Borland *et al.*, 2011). In effect, the proposal is to push the proportional contribution of CO_2_ fixation in the light into the vicinity of 50–60%, a proportion that large surveys of CAM plants have revealed as being uncommon in the natural environment (Winter and Holtum, 2002; Crayn *et al.*, 2004; Silvera *et al.*, 2010).

As in *A. angustifolia*, 24h CO_2_ exchange by the most highly productive CAM species grown in commercial plantations in warm and temperate subtropical dry-land environments, *A. tequilana*, *O. ficus-indica*, *A. salmiana* and *Agave mapisaga*, is dominated year round by nocturnal CO_2_ fixation (Nobel, 1996*a*; Nobel *et al.*, 1992; Pimienta-Barrios *et al*., 2001, 2006). In *A. tequilana*, environmental factors that limited growth and productivity in the field were water during the cool dry winter and PAR during the warm wet summer (Nobel and Valenzuela, 1987), not CO_2_ fixation in the light. Under these field limitations, a shift towards an increase in the capacity for CO_2_ fixation in the light is unlikely to result in significantly increased productivity. Rather, productivity might be expected to be higher if plants were grown at sites that were less cloudy in the summer and more moist in the winter.

Proposals to grow *Agave* as biofuel feedstocks in seasonally dry regions have emphasized their suitability for so-called marginal lands (Borland *et al.*, 2009; Somerville *et al.*, 2010; Davis *et al.*, 2011). Land that is marginal for growing traditional crops may well support high growth rates of water-use efficient, high-temperature-tolerant CAM species, but it remains to be seen whether *Agave* can produce commercially relevant yields on truly marginal lands that are nutrient poor and severely water limited.

## Abbreviations:

CAM: crassulacean acid metabolism
PFD: photon flux density.
